# Effectiveness of a probiotic combination on the neurodevelopment of the very premature infant

**DOI:** 10.1038/s41598-023-37393-6

**Published:** 2023-06-26

**Authors:** Benjamin James Baucells, Giorgia Sebastiani, Leyre Herrero-Aizpurua, Vicente Andreu-Fernández, Elisabet Navarro-Tapia, Oscar García-Algar, Josep Figueras-Aloy

**Affiliations:** 1 Neonatology Service Hospital Clínic-Maternitat, ICGON, BCNatal, Carrer Sabino Arana 1, 08028 Barcelona, Spain; 2 Grup de Recerca Infància i Entorn (GRIE), Institut d’investigacions Biomèdiques August pi i Sunyer (IDIBAPS), Barcelona, Spain; 3 Paediatrics Service, Hospital del Mar, Barcelona, Spain; 4 Biosanitary Research Institute, Valencian International University (VIU), Valencia, Spain; 5 Faculty of Health Sciences, Valencian International University (VIU), Valencia, Spain

**Keywords:** Paediatric research, Neurodevelopmental disorders, Nutrition

## Abstract

Probiotics have shown a benefit in reducing necrotising enterocolitis in the premature infant, however the study of their effect on premature neonates’ neurodevelopment is limited. The aim of our study was to elucidate whether the effect of *Bifidobacterium bifidum* NCDO 2203 combined with *Lactobacillus acidophilus* NCDO 1748 could positively impact the neurodevelopment of the preterm neonates. Quasi-experimental comparative study with a combined treatment of probiotics in premature infants < 32 weeks and < 1500 g birth weight, cared for at a level III neonatal unit. The probiotic combination was administered orally to neonates surviving beyond 7 days of life, until 34 weeks postmenstrual age or discharge. Globally, neurodevelopment was evaluated at 24 months corrected age. A total of 233 neonates were recruited, 109 in the probiotic group and 124 in the non-probiotic group. In those neonates receiving probiotics, there was a significant reduction in neurodevelopment impairment at 2 years of age RR 0.30 [0.16–0.58], and a reduction in the degree of impairment (normal-mild vs moderate-severe, RR 0.22 [0.07–0.73]). Additionally, there was a significant reduction in late-onset sepsis (RR 0.45 [0.21–0.99]). The prophylactic use of this probiotic combination contributed to improving neurodevelopmental outcome and reduced sepsis in neonates born at < 32 weeks and < 1500 g.

## Introduction

Probiotics are “live microorganisms which, when administered in adequate amounts, confer a health benefit on the host”^1^. Preterm neonates, below 32 weeks gestational age and under 1500 g birthweight, show dysbiosis produced by a delay in microbiota acquisition^2^. Dysbiosis is combined with a substantial difference in microbiota composition, with lower numbers of *Lactobacillus* and *Bifidobacterium* (considered protective microorganisms) and risen *Enterobacteriaceae*, containing potential pathogens such as *Escherichia coli* and *Klebsiella*^2^. Delayed human milk introduction, early antibiotic therapy, caesarean delivery, and total parenteral nutrition, predispose to microbiota imbalance^3^. In premature infants, dysbiosis could dysregulate proinflammatory and protective factors, increasing the risk of necrotising enterocolitis (NEC), and raising mortality^3^. Nowadays, numerous publications show the benefit of using probiotics to prevent NEC and late onset sepsis (LOS), especially when combining strains of *Bifidobacterium* and *Lactobacillus*^4–6^.

A premature infant is faced with a variety of neurological complications and potential sequelae, including psychomotor and neurological delay, with consequences beyond cerebral palsy, hearing loss and blindness^7,8^. With regards to neurodevelopment, the gut-brain axis is interconnected through endocrine, neural and immune pathways^9–11^. Microbiota, with the intrinsic capability of processing indispensable metabolites, regulating pathogenic microorganisms, and modulating the immune response^3,12^—for instance by reducing gut permeability to the Lipopolysaccharide (LPS, pro-inflammatory factor)^3,12^—could play a critical role in the brain-gut axis^11^. Substances such as the brain-derived neurotrophic factor (BDNF), neurotrophins, or interleukin-6, involved in neuroinflammation and neurodevelopment^13^, can be regulated by changes in the microbiota^14^. Furthermore, the BDNF, the nerve growth factor and neurotrophins 3 and 4, promote neurone survival and diminish apoptosis in the central and peripheral nervous systems, being of vital importance in the pre and postnatal development of the brain^15,16^. Despite all the previous findings, little has been published regarding the effect of probiotics on the premature infant neurodevelopment, with recent published studies finding no benefit^17–21^.

The objective of this study was to evaluate the effect of combining two probiotics (*Bifidobacterium bifidum* NCDO 2203 and *Lactobacillus acidophilus* NCDO 1748) in the neurodevelopment of preterm neonates below 32 weeks’ gestation and a birthweight under 1500 g. This probiotic combination has shown to be safe and beneficial in premature neonates in the prevention of NEC^4^. We hypothesised that this mixture would contribute to better neurodevelopmental outcomes of preterm neonates when assessed at 24 months corrected age. Secondarily, these probiotics could reduce NEC, LOS, intraventricular haemorrhage and neonatal mortality in accordance with previous studies^4,6^.

## Materials and methods

### Design

Quasi-experimental, unicentric cohort study, where the probiotic intervention was implemented for one group (born 2014–2016) and compared to a control group (born 2018–2019) with follow-up and neurodevelopment evaluation with neuropsychological tests at 24 months corrected age. Quasi-experimental studies aim to evaluate interventions but do not use randomisation. Given the described high risk of cross-contamination and ethical concerns^22,23^, we sought a sequential design with consecutive recruitment, without randomisation, with a washout period between both groups (year 2017)^22^. Using “Power and Sample Size Calculations”^24^ version 3.0.14, considering an alpha risk of α = 0.05 and a beta risk of β = 0.2 in a bilateral contrast, a minimum of 90 subjects in each group were required to detect a statistically significant difference between groups, where for the control group the proportion of some neurodevelopmental alteration was expected to be at least 0.4 and for the group treated with probiotics at least 0.2. The accepted loss-to-follow-up rate was 20%, which meant the subjects needed were 109 in both groups. Expecting higher mortality in the control group, as probiotics have been shown to reduce mortality^4^, we increased the control group by 10% to ensure having enough sample size at the 24-month evaluation. The values of α and β were selected in line with standard scientific publications^25,26^ ensuring adequate significance with optimum statistical power.

### Patients and intervention

Infants born below 32 weeks gestational age and birth weight under 1500 g cared for at BCNatal Hospital Clínic (tertiary neonatal unit) in Barcelona between January 2014 and December 2019. Patients received a daily dose of 6 × 10^9^ UFC Infloran® -Berne, Switzerland- (*Bifidobacterium bifidum* NCDO 2203 and *Lactobacillus acidophilus* NCDO 1748) from 7 days of life until reaching a postmenstrual age of 34 weeks or discharge. The probiotic mixture was provided in capsules, which were opened, dissolved in water and given orally or via nasogastric tube, according to the feeding regime of each neonate (breast milk, donor milk or formula). Where concerns for NEC or LOS were present, probiotics were stopped and reinstated once enteral feeds were recommenced. The study was conducted in accordance with the Declaration of Helsinki and approved by the Ethics Committee in Drug Research of Hospital Clínic (HCB/2021/0454-April 2021). Informed consent was obtained from all subjects involved in the study. The unit had an existing standardised nutrition protocol.

### Inclusion and exclusion criteria

**Figure 1:**
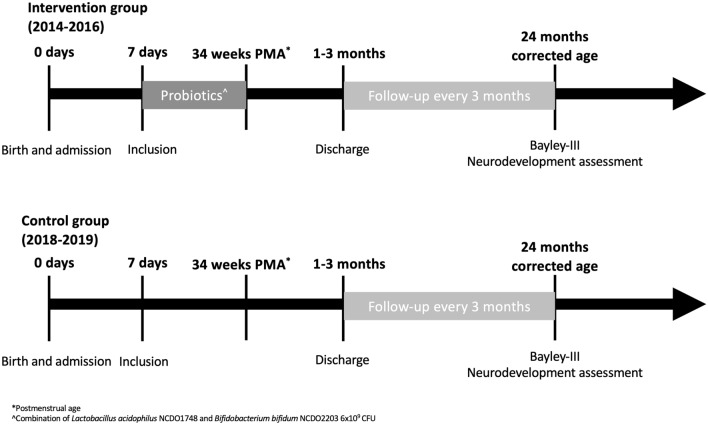
Brief depiction of the conducted study, group intervention, follow-up and main analysed outcome.

All neonates below 32 weeks gestational age and 1500 g who fulfilled the inclusion criteria were recruited, independently of their method of delivery. Those born between years 2014 and 2016 were allocated to the intervention group and received the probiotic combination (Fig. 1). The control group was created using neonates born between years 2018 and 2019. In this group we included all neonates born under 32 weeks, and 1500 g and surviving beyond 7 days of life (age when probiotics were introduced in the previous cohort).

All neonates presenting with suspected congenital anomalies, inborn errors of metabolism, or genetic defects were excluded. Infants with a suspected syndrome, or who had suffered events beyond the neonatal period, not related to prematurity, that could entail impairment in neurodevelopment (severe cranioencephalic trauma, oncological process, meningitis, or exposure to toxic substances) were also excluded.

### Outcomes

All patients underwent a standardised follow-up program for high-risk premature infants at our centre, up until 24 months corrected gestational age. At that stage, a Bayley-III scale test^27^ was included in an extensive examination performed by independent assessors, blinded to group allocation. After this evaluation, patients were divided into four categories according to their degree of neurodevelopment: survival without neurodevelopment impairment (normal neurodevelopment), mild impairment, moderate impairment, or severe impairment^28^. Mild impairment was considered if they had muscle tone changes, impaired fine or gross motor coordination, Bayley scale score between 71 and 84, moderate behaviour disorders or mild visual disability. Moderate impairment was diagnosed when suffering from spastic diplegia, hemiplegia, seizures (non-febrile), Bayley scores between 50 and 70, severe behaviour disorders, moderate visual disability or mild-moderate hypoacusis. Severe impairment was attributed to subjects with spastic quadriplegia, choreoathetosis, ataxia, Bayley score < 50, blindness or severe hypoacusis.

The secondary outcomes were the incidence of NEC, all-cause mortality, LOS, retinopathy of prematurity, intraventricular haemorrhage and intensive care length of stay. We defined NEC as those cases fulfilling the stage II or above of the modified Bell’s Criteria^29^. LOS was defined as a positive blood culture beyond 72 h of life. Close monitoring was implemented for side effects of the probiotic administration and probiotic sepsis.

### Statistical analysis and measurement of treatment effect

IBM SPSS Statistics 27.0.1.0 was used for the statistical analysis. Non-parametrical analysis with a double-sided Mann–Whitney U test was calculated for all continuous variables, whilst for categorical variables the chi-square test was applied. In all cases, 0.05 was considered the threshold of statistical significance. The relative risk (RR) and 95% confidence intervals were determined for dichotomous variables. When encountering significant differences, the number needed to treat (NNT) was calculated.

### Consent statement

The study was conducted in accordance with the Declaration of Helsinki and approved by the Ethics Committee of Hospital Clínic (HCB/2021/0454- April 2021). Informed consent was obtained from parents or legal guardians for all subjects involved in the study.

## Results

### Baseline characteristics

**Figure 2:**
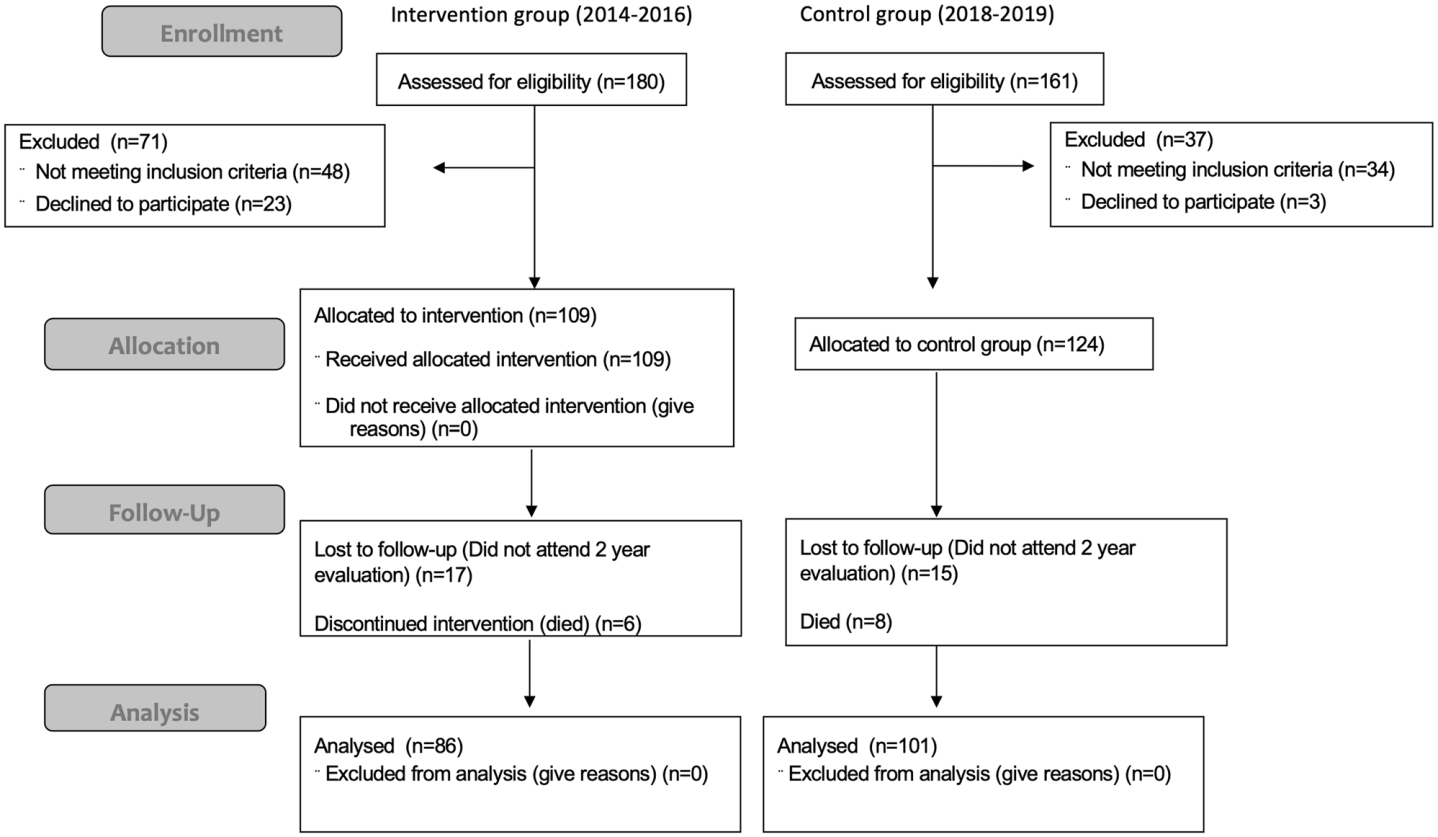
Flowchart showing study selection of subjects.

A total of 233 neonates were included in the analysis after fulfilling all inclusion and exclusion criteria: 109 neonates in the probiotic group and 124 in the control group (Fig. 2).

Preterm infants that received probiotics were significantly more mature than those neonates that did not receive probiotics (29 weeks vs 28.5 weeks), however, this difference had no clinical relevance. There was also a moderately higher incidence of multiple gestation births and preeclampsia in the probiotic group. There were no other significant differences between groups at baseline (Table 1).

**Table 1:** Baseline characteristics of the probiotic and non-probiotic cohort.

Variable	Probiotics (n = 109)	No Probiotics (n = 124)	Significance
Gestational age (weeks)	29 (27.9–30.3)	28.5 (26.9–30.0)	p = 0.034*
Weight (g)	1040 (909–1260)	1065 (830–1280)	NS
Maternal age (years)	35(29–38)	34 (30–37)	NS
Multiple gestation	50 (45.9%)	41 (33.1%)	p = 0.046*
Antenatal steroids (full course)	80 (73.4%)	95 (76.6%)	NS
Antenatal steroids (partial course)	25 (22.9%)	25 (20.2%)	NS
Sex (men)	58 (53.2%)	54 (43.5%)	NS
Maternal hypertension	27 (24.8%)	16 (12.9%)	p = 0.020*
Labour (caesarean)	73 (67%)	75 (60.5%)	NS
Chorioamnionitis	29(26.6%)	30 (24.8%)	NS
Advanced resuscitation	18 (16.7%)	18 (14.8%)	NS
Umbilical artery pH	7.27 (7.21–7.32)	7.26(7.19–7.30)	NS
Apgar, 5 min (median and IQR)	9 (8–10)	9 (8–10)	NS
Exclusive breastfeeding	65.4%	64.5%	NS
Mixed feeding	29 (27.1%)	40 (33.3%)	NS
Formula feeding	8 (7.5%)	3 (2.5%)	NS

Variables expressed as median and interquartile range or percentage, where applicable. Statistical analysis was performed using non-parametric U of Mann Whitney, with a degree of significance at 0.05. Antenatal steroids: administration of corticosteroids (betamethasone) to induce lung maturation prior to preterm birth, and to reduce respiratory distress; 1 dose is considered partial, and 2 doses are considered a full course. Chorioamnionitis: Infection of the placenta and the amniotic fluid. Mixed feeding: Combination of breast milk and formula feeding. Advanced resuscitation: Need for intubation, cardiac compressions, or adrenaline in labour suite.

*Statistical significance.

### Effect of probiotics on neurodevelopment

Of all the included patients, 86(78%) of infants receiving the probiotic combination, and 101(80%) infants of the non-probiotic cohort underwent a full 2-year review. There was a significant increase in survival without neurodevelopment impairment in the probiotic cohort at 24 months corrected age, RR 0.30 [0.16–0.58], NNT 3.85 [2.6–7.1]. The probiotic intervention also led to an improvement in overall Bayley-III Scale scores with a statistically significant difference in language with median scores of 94 [89–100] vs 88.5 [77–97] p = 0.006 respectively.

The degree of impairment was also less severe in the probiotic group (normal-mild vs moderate-severe, RR 0.22 [0.07–0.73]), NNT 8.1 [4.8–25.9]. The effect was still significant when comparing mild and moderate impairment with RR 0.37 [0.17–0.83] and RR 0.21 [0.05–0.94] subsequently. The overall incidence of severe neurodevelopment impairment was low in both groups, with no statistically significant differences. All neurodevelopment outcomes can be found in Table 2. Neurodevelopmental outcomes did not vary after analysing effect of preeclampsia and gestational age.

**Table 2:** Neurodevelopmental outcomes at the 2-year analysis.

	Probiotics	No Probiotics	Relative risk [95% CI]	χ^2^	p-value
Neurodevelopment at 2 years					P < 0.001*
Normal	76/86	63/101			
Mild impairment	7/86	22/101	0.37 [0.17–0.83]	6.59	p = 0.0159*
Moderate impairment	2/86	11/101	0.21 [0.05–0.94]	5.27	p = 0.0407*
Severe impairment	1/86	5/101	0.24 [0.03–1.97]	2.15	p = 0.1821
Normal vs impaired			0.30 [0.16–0.58]	16.45	p = 0.0003*
Normal-mild vs moderate-severe			0.22 [0.07–0.73]	7.77	p = 0.0013*
Bayley-III					
Mental	99 (95–104)	95 (85–105)			p = 0.244
Motor	97 (91–103)	97 (85–107)			p = 0.323
Language	94 (89–100)	88.5 (77–97)			p = 0.006*

*Statistical significance.

### Secondary outcomes

When focusing on secondary outcomes (Table 3), probiotics did not affect incidence of NEC, overall mortality beyond 7 days of life, intraventricular haemorrhage, retinopathy of prematurity, nor periventricular leukomalacia.

**Table 3:** Outcomes of the probiotic and non-probiotic group from birth up until the 2-year analysis.

Outcome	Probiotics	No probiotics	Relative risk [95% CI]	p-value
Respiratory distress syndrome	43/109	45/124	1.007 (0.78–1.51)	P = 0.620
Late-onset sepsis	8/109	20/124	0.45 [0.21–0.99]	p = 0.040*
Intraventricular haemorrhage	22/109	32/124	0.78 [0.49–1.27]	p = 0.311
Retinopathy of prematurity	28/103	38/116	0.84 [0.55–1.27]	p = 0.370
Patent ductus arteriosus	35/109	42/124	0.95 [0.66–1.37]	p = 0.776
Necrotising enterocolitis	3/109	3/124	1.14 [0.23–5.52]	p = 0.873
Death beyond 7 days of life	6/109	8/124	0.85 [0.30–2.38]	p = 0.761
Periventricular leukomalacia	6/103	2/116	3.38 [0.69–16.4]	p = 0.107

*Statistical significance.

There was a significant reduction in LOS, RR 0.45 [0.21–0.99], NNT 11.4 [5.8–200.7]. Additionally, there was a statistically significant reduction in the intensive care length of stay in the probiotic cohort 9 [6–15] vs 14 [7–41.5] days (p < 0.001). During the study, there were no cases of probiotic sepsis nor side effects.

## Discussion

Recent literature demonstrated the beneficial effects of probiotics on NEC and LOS in very low birth weight neonates when used in the first weeks of life^4–6,30^. However, neurodevelopment benefits of probiotic use have not been clearly demonstrated^21,31,32^. The lack of effect of probiotics in neurodevelopment in previous studies (Table 4) could have resulted from varying factors.

**Table 4:** Comparison of the previous existing studies analysing the use of probiotics and premature infant neurodevelopment*.*

Study	Probiotic used	Neurodevelopment assessment	Main results
Jacobs et al.^17^	*Bifidobacterium infantis* BB-02 96579 30 × 10^6^*, Streptococcus thermophilus* TH-4 15957 350 × 10^6^ and *Bifidobacterium lactis* BB-12 15954 350 × 10^6^ (ABC Dophilus Probiotic Powder for Infants, Solgar USA)	Composite of (at least one) at 2–5 years corrected age: Bayley-III < 77.5 or WPPSI-III < 2 standard deviations CP with GMFCS 2–5 Deafness requiring hearing aid or cochlear implant Blindness 6/60 better eye	No difference in survival free of neurodevelopment impairment RR 1.01 [0.93–1.09]. Deafness lower in probiotic group (0.6% vs 3.4%)
Sari et al.^18^	*Lactibacillus sporogenes* 350 × 10^6^(DMG ITALIA SRL, Rome Italy)	Composite outcome of at last one at 18–22 months corrected age: Bayley-II < 70 PDI < 70 Deafness needing hearing aids in both ears Blindness with no useful vision in either eye CP (abnormal one in 1 limb)	There was no significant difference in growth and neurodevelopmental outcomes (p = 0.788) between the two groups
Romeo et al.^19^	*Lactobacillus reuteri* ATCC 55730 1 × 10^8^ or *Lactobacillus rhamnosus* ATCC 53103 6 × 10^9^	Hammersmith score of < 73 (suboptimal) at 12 months corrected age	No statistical differences were observed in the incidence of suboptimal scores in both probiotic groups (p > 0.05). Statistically significant difference of suboptimal scores (p < 0.05) in the control group vs both probiotic groups
Chou et al.^20^	*Lactobacillus acidophilus* 10^9^ and *Bifidobacterium infantis* 10^9^ (Swiss Serum and Vaccine Institute, Berne, Switzerland)	Composite of (at least one) at 3 years corrected age: Bayley-II < 70 PDI < 70 Bilateral Blindness Hearing impairment > 55 dB in both ears CP requiring ambulatory assistance	No significant differences in growth or in any of the neurodevelopmental and sensory outcomes between the 2 groups
Akar et al.^21^	*Lactobacillus reuteri 10*^*8*^ (Biogaia AB, Stockholm, Sweden)	Composite outcome of at last one at 18–24 months corrected age: Bayley-II < 70 PDI < 70 Deafness needing hearing aids in both ears Blindness with no useful vision in either eye CP (abnormal tone in 1 limb)	Neurodevelopment impairment did not differ between groups Probiotic 37/124 vs Non-probiotic 37/125 p = 0.96

WPPSI (Weschler Preschool and Primary Scale of Intelligence), CP (Cerebral Palsy), PDI (Psychomotor development index.

For Jacobs et al.^17^, Sari et al.^18^, Romeo et al.^19^ and Akar et al.^21^ the use of different strains could explain their absence of benefits. As described, probiotics encompass a wide range of microorganisms that have completely different effects according to strain, dose and even site^33^. Different timing of the supplementation, as well as variations in gestational age or birth weight of the new-borns, could also influence the effectiveness of the treatment. Current evidence shows the best efficacy is achieved when combining probiotics^3,4^.

Our study is the first to show benefit with a significantly higher proportion of infants having normal neurodevelopment outcomes when given probiotics. Furthermore, there was a reduction in the length of stay in intensive care by almost half in these infants.

Traditionally, mild-moderate neurodevelopment impairment has seldom been considered in studies of premature outcomes^7,17,18,20,21^. Our study considered smaller degrees of impairment given their potential major impact on later quality of life. A broader spectrum of neurodevelopment evaluations could have highlighted a more subtle effect of probiotics, and impacted our results, compared to more restrictive criteria of other authors^17,18,20,21^. Moreover, mild and moderate neurodevelopment impairment are much more prevalent than severe impairment^34^.

Current literature suggests that neurocognitive disabilities manifest around 5–6 years of age^8^ and therefore 12-month evaluations could mask the true impact of probiotics. The inclusion up until 5 years corrected age of Jacobs et al.^17^ could help clarify such an effect. However, the loss of participants underpowered their study. Likewise, the lack of statistical power in the study by Chou et al.^20^ could explain the differences encountered with our study, despite the use of similar combinations of probiotics.

Finally, differing inclusion criteria with varying birthweights and gestation, also play a role in the findings of each study.

In short, key strong points of our study are the use of a probiotic combination with well-established benefits in preterm neonates^5,6,35^, the extensive follow-up and evaluation programme of premature infants at risk, and a broader analysis of neurodevelopmental outcomes. Unfortunately, the lack of randomisation and the sequential nature of the study could introduce bias in the results presented. Also, establishing an analysis at 24 months corrected age could mask the full impact of probiotics on neurodevelopment. Although there was higher preeclampsia in the probiotic group, and possible exposure to magnesium sulphate –a widely used treatment for maternal hypertension and proposed therapy for improving neurodevelopment outcome^36^-, once adjusted by maternal hypertension differences in neurodevelopment impairment were sustained between groups. An overall low incidence of severe neurodevelopment impairment in both our study groups could have made achieving statistical significance difficult. Nonetheless, the reduction of severe cases of neurodevelopment impairment in the probiotic group compared to the control group has clinical relevance. Lastly, despite borderline statistically significant differences in gestational age between groups, we considered a difference of 2 days in gestational age not to have clinical significance.

When centring on the explanations of the effect of probiotics on the neurodevelopment, the existence of the brain-gut axis has been proven to be of paramount importance^9–11^. Dysbiosis can contribute to the increase of gut permeability causing a rise in bacterial translocation and LPS, directly implicated in chronic inflammation, leading to an upscaling of pro-inflammatory cytokines such as interleukine-6 and tumour necrosis factor α and subsequent cortisol activation^10,11,13^. *Lactobacillus helveticus* combined with *Bifidobacterium longum* have shown a decrease in hippocampal apoptosis in rats exposed to LPS^37^, as well as reducing serum cortisol -main stress hormone- levels in humans and rats^38^. *Bifodobacterium bifidum* and *Lactobacillus salivarius* administered in the prenatal period, decreased LPS and impacted foetal gut microbiota^39^. Cortisol has been found to influence the secretion of BDNF. Exposure to postnatal steroids in premature infants has been linked to an increased risk of cerebral palsy and neurobehavioural changes^40^. As pointed out by Bailey et al.^41^, stress can induce dysbiosis and a reduction in native *Lactobacillus* in human gut, which is then related to an increase in interleukin-6 and posterior anxiety-like patterns of behaviour. Studies in mice have shown that the correction of dysbiosis in adulthood did not normalise behavioural patterns and neuroregulation as it did if corrected in early stages of life^42^. Therefore, correction should be within this critical window to ensure the long-term benefits in neurodevelopmental programming, thus reinforcing the need for probiotic implementation early in the timeline of the developing premature brain.

The study of Kim et al.^43^ showed the ability of *Bifidobacterium bifidum* BGN_4_ to increase BDNF expression and reverse apoptosis in aging mouse models. As proposed by Liu et al.^16^, BDNF could have a major role in Attention Deficit Hyperactive Disorder (ADHD) and other psychiatric disorders, which, as reflected in the EPIC study group, premature infants have a higher risk of manifesting^44^. Additionally, premature infants have lower numbers of *Bifidobacterium bifidum* compared to term neonates^45^. Although it should be considered cautiously, the study of Wang et al.^46^ hinted that during the administration of *Bifidobacterium bifidum* (Bf-688) children with ADHD showed improvement in symptoms. The administration of *Lactobacillus rhamnosus* (JB-1) to mice induced a changed expression of GABAB1b and GABAA⍺2 receptor subunits mRNA^47^. Interestingly, vagotomy impeded the effects of the probiotic on the GABA_A⍺2_ subunit, enhancing the theory that the vagus nerve is important in the brain-gut axis, stress and anxiety.

A reduction in the total days of intensive care, seen in the probiotic cohort, and transfer to a lower level of care, with a reduction of painful procedures and overall stress, could also contribute to better neurobehavioural development^48^.

Alcon-Giner et al. have published a study on the metabolome of premature infants supplemented with the same mixture used in our study. They showed that the supplemented preterm infants had a lower abundance of potential pathobionts, more typical of the preterm gut, which have previously been linked to NEC and LOS^49^. Furthermore, the *B. bifidum* Infloran® strain was able to metabolise specific human milk oligosaccharides. This fact not only facilitates digestion and improves nutrition in the preterm neonates, but the human milk oligosaccharide degradation by-products (acetate and lactate) inhibit the growth of some pathogenic bacteria, reinforce the intestinal barrier, and have anti-inflammatory effects. Therefore, hinting the possible neuroprotective properties for preterm neonates. The authors also observed that *B. bifidum* Infloran® strain was able to persist in the gut for more than 50 days after the end of treatment.

On the other hand, the reduction in late-onset sepsis by probiotics was concordant with previous findings^4,30^. Said outcome could also help explain the effects in neurodevelopment. Studies in rat models have manifested that infection can cause alterations in the expression of BDNF. Probiotics such as *Bifidobacterium longum* can help palliate such an effect and restore BDNF expression^10^. Moreover, the use of *Lactobacillus plantarum ZLP001* was shown to combat the increase in gut permeability caused by enterotoxigenic *Escherichia coli* diminishing proinflammatory cytokines in piglets^50^. Exposure to antibiotics is well known to diminish beneficial microbiota such as *Lactobacillus* and cause dysbiosis. Hence, a reduction in the number of septic episodes would decrease the use of antibiotics. However, the rise in antibiotic stewardship might have contributed to decrease initial antibiotic exposure, affecting our results.

Finally, although the probiotic combination used has been shown to reduce NEC^51^, our lack of statistical differences in NEC could be justified by a traditionally low incidence in our unit (yearly incidence of NEC < 3%), combined with a high use of expressed breast milk and donor milk, with a very low prevalence of formula-exposed preterm neonates. There has been extensive research in the use of breast milk for the reduction of NEC. As shown by a recent meta-analysis^52^, exclusive breast feeding is superior to formula and mixed feeding in randomised controlled trials.

To the best of our knowledge, this is the first study to prove the benefit of a probiotic mixture in premature infant neurodevelopment. However, since the bigger impact of prematurity in neurodevelopment is truly shown in later stages of life, further studies are needed in older infants to understand the full scope of said intervention, opening a new field of studies in probiotics and the premature infant. It would also be interesting to conduct studies with postbiotics in this area. Postbiotics not only have more advantages than probiotics when used in these vulnerable populations but also have proven safety, anti-inflammatory, immunomodulating and antimicrobial action. These characteristics are of great help in very preterm infants, where intestinal permeability and inflammation is evident^53^.

## Conclusions

The prophylactic administration of *Bifidobacterium bifidum* NCDO2203 combined *with Lactobacillus acidophilus* NCDO1748 contributed to improve neurodevelopmental outcome and reduce LOS in neonates born at less than 32 weeks and less than 1500 g. More clinical trials would be needed to corroborate these findings and to explain the mechanisms by which reversing dysbiosis may improve neurological outcomes.

## Data Availability

The datasets generated and/or analysed during the current study are not publicly available due patient confidentiality but are available from the corresponding author on reasonable request.

## Abbreviations

ADHD: Attention deficit and hyperactive disorder
BDNF: Brain derived neurotrophic factor
CP: Cerebral Palsy
LOS: Late-onset sepsis
LPS: Lipopolysaccharide
NEC: Necrotising enterocolitis
NNT: Numbers needed to treat
PDI: Psychomotor development index
RR: Relative risk
WPPSI: Weschler Preschool and Primary Scale of Intelligence
